# 

**Published:** 1858-01

**Authors:** 


### THE SANITARY REVIEW.

CONTENTS of No. XII.

Registrations of Disease in England . . . . . . 317
The Social Epidemic . . . . . . 327
Metropolitan Hygiene in the Past . . . . . . 335

**EPITOME OF SANITARY LITERATURE:**

Miscellaneous Works . . . . . . 351

**ORIGINAL COMMUNICATIONS:**

The Medical Pilgrim's Progress. By B. W. Richardson, M.D. . 353
Notes on Cases of Hydrophobia. By William Pickells, M.D. . 371

**SANITARY AND SOCIAL SCIENCE.**

*Reports on Cities, Towns, and Districts.*—Cardiff . . . 392
Food Grains of India . . . . . . 395
Local Boards of Health in England . . . . 397

**PROGRESS OF EPIDEMICS in Forty-one Towns and Districts, contributed specially, by the Staff of Local Observers** . . . 398

**SANITARY LEGISLATION** . . . . . 416

**TRANSACTIONS of the EPIDEMIOLOGICAL SOCIETY of LONDON:**

Introductory Address delivered at the Opening of the Session, 1857-58. By B. G. Babington, M.D., F.R.S. . . . . 73
The Study of Epidemic Diseases; Illustrated by the Pestilences of London. By E. H. Greenhow, M.D. . . . . 77

